# Biliary drainage surgery before or after 3 months of life *versus* primary liver transplantation in children with biliary atresia: comparative cohort study

**DOI:** 10.1093/bjsopen/zrac175

**Published:** 2023-03-23

**Authors:** Juri Fuchs, Chaima Mrad, Emmanuel Gonzales, Dior Ndiaye, Virginie Fouquet, Géraldine Héry, Catherine Baujard, Florent Guérin, Sophie Branchereau

**Affiliations:** Department of General, Visceral, Paediatric and Transplantation Surgery, University of Heidelberg, Heidelberg, Germany; Department of Paediatric Surgery, Paris-Saclay University, Assistance Publique-Hôpitaux de Paris, Bicêtre Hospital, Paris, France; Department of Paediatric Surgery, Paris-Saclay University, Assistance Publique-Hôpitaux de Paris, Bicêtre Hospital, Paris, France; Department of Paediatric Hepatology, Paris-Saclay University, Assistance Publique-Hôpitaux de Paris, Bicêtre Hospital, Paris, France; Department of Paediatric Surgery, Paris-Saclay University, Assistance Publique-Hôpitaux de Paris, Bicêtre Hospital, Paris, France; Department of Paediatric Surgery, Paris-Saclay University, Assistance Publique-Hôpitaux de Paris, Bicêtre Hospital, Paris, France; Department of Paediatric Surgery, Paris-Saclay University, Assistance Publique-Hôpitaux de Paris, Bicêtre Hospital, Paris, France; Department of Anaesthesiology and Intensive Care, Paris-Saclay University, Assistance Publique-Hôpitaux de Paris, Bicêtre Hospital, Paris, France; Department of Paediatric Surgery, Paris-Saclay University, Assistance Publique-Hôpitaux de Paris, Bicêtre Hospital, Paris, France; Department of Paediatric Surgery, Paris-Saclay University, Assistance Publique-Hôpitaux de Paris, Bicêtre Hospital, Paris, France

## Abstract

**Background:**

Early biliary drainage surgery (BDS; Kasai) is associated with longer transplant-free survival in biliary atresia. However, evidence is lacking on whether an age limit can be established at which liver transplantation should be performed as first-line treatment for children with a delayed diagnosis of biliary atresia. The aim of the current study was to compare the outcome of a large cohort of children with biliary atresia who underwent BDS after 90 days of life with those who underwent early BDS (before 90 days) and those who did not receive BDS and were directly referred for primary liver transplantation.

**Methods:**

All patients with biliary atresia treated at Bicêtre, Paris-Saclay University Hospital between 1995 and 2017 were analysed in this STROBE-compliant study. Three groups were defined: BDS before 90 days of life (early BDS); BDS after 90 days of life (late BDS); and patients without BDS who were referred for primary liver transplantation (no BDS). Patient characteristics, overall survival, and native liver survival were compared.

**Results:**

Of 424 children with biliary atresia, 69 patients (16 per cent) were older than 90 days when they underwent BDS. Twenty-five patients had no BDS and were referred for primary liver transplantation (6 per cent). The main reason for not performing BDS was manifest portal hypertension (18/25). Two- and 5-year transplant-free survival were significantly higher in patients with late BDS compared with no BDS (53.5 *versus* 12.0 per cent respectively for 2-year data and 30.4 *versus* 4.0 per cent respectively for 5-year data, *P* < 0.001). Five- and 10-year overall survival did not differ between early BDS (92 and 91 per cent respectively), late BDS (88 and 83 per cent respectively) and no BDS (80 and 80 per cent respectively, *P* = 0.061).

**Conclusion:**

Age alone should not routinely be considered a contraindication to BDS in patients older than 90 days. Liver transplantation in infancy (less than 12 months) could be avoided in 88 per cent of cases with late diagnosis of biliary atresia and is delayed significantly even when BDS is performed after 3 months. Overall survival is at least equal to patients who are referred for primary liver transplantation.

## Teaser

Evidence is lacking on whether an age limit can be established at which liver transplantation (LT) should be performed as first-line treatment for children with a delayed diagnosis of biliary atresia (BA). Our study results show that LT in infancy (less than 12 months) can be avoided in 88 per cent of cases with late diagnosis of BA and is delayed significantly even when Kasai is performed after 3 months. Late Kasai can be recommended even in cases of late diagnosis of BA.

## Introduction

Biliary atresia (BA) is a rare neonatal liver disease with heterogeneous aetiology and clinical evolution and remains fatal if untreated. While long-term native liver survival can be achieved in some patients when performing biliary drainage surgery (BDS; mostly Kasai portoenterostomy (PE)), approximately 80 per cent of patients born in Western countries require liver transplantation (LT) when BDS has failed, could not be performed, or other hepatological complications arise^1^. Young age at diagnosis and at BDS has been repeatedly shown to be a predictor of better outcomes, in particular longer native liver survival^2–4^. However, due to its rarity and the difficulty in diagnosing BA, a significant proportion of patients are diagnosed late. Previous studies found that the prognosis for affected infants deteriorates significantly at around 3 months of age^2,5^. Given the considerable improvements in safety and long-term success of paediatric LT, the question arises of whether BDS should be performed at all in children with BA older than 90 days^6,7^. Some authors advocate direct referral for LT as first-line treatment for these patients, arguing that previous BDS adds surgical risks and has ramifications for LT^7–9^. Others emphasize the remarkable success rates of BDS even at older ages and the importance of prolonging native liver survival in view of persistent organ shortage for paediatric patients, particularly for infants (less than 12 months) with a bodyweight of less than 10 kg^5,6,10–12^.

The aim of the current study was to compare the outcome of a large cohort of children with BA who underwent BDS after 90 days of life with those who underwent early BDS (before 90 days) and those who did not receive BDS and were directly referred for primary LT.

## Methods

### Ethics

This study was conducted in accordance with the guidelines of the revised U.N. Declaration of Helsinki in 1975 and its latest amendment in 2013 (7th revision). In addition, it was approved by the *Commission Nationale de l'Informatique et des Libertés* (CNIL; approval number 1874973).

### Study design, patient population, and sample size calculation

In adherence with the STROBE guidelines^13^, a retrospective, comparative single-centre cohort study was conducted. All paediatric patients who were treated for BA at Bicêtre, Paris-Saclay University Hospital between 1 January 1995 and 31 December 2017 were eligible. Three subgroups were defined as follows: early BDS, patients with BDS (mostly Kasai PE) performed before 90 days of life; late BDS, patients with BDS performed after 90 days of life; and no BDS, patients without BDS who were referred for primary LT.

### Data acquisition, management, and follow-up

Data were extracted from a prospectively maintained database that is run by a dedicated data management team at the Bicêtre, Paris-Saclay University Hospital. In addition to those data, missing information was collected from the medical administration software of Bicêtre, Paris-Saclay University Hospital. All patients were included in a standardized follow-up programme. After discharge, a first check-up was performed 1 month later. In cases without complications, the next appointments were performed 3 and 6 months after discharge and were repeated at 6 months intervals. Closer follow-up with, in some cases, even weekly appointments, was performed on an individual basis depending on patient status. The check-ups consisted of clinical examinations, blood tests, and abdominal ultrasonography. Additional investigations were performed as indicated.

### Outcome measures and statistical analyses

The main outcome measures of the study were native liver survival and overall survival, calculated according to the Kaplan Meier estimates. Additional investigated outcomes were: postoperative morbidity, with major complications defined as adverse effects necessitating reoperation or interventions, corresponding to complications greater than or equal to grade IIIa according to the Clavien–Dindo classification^14^; success of BDS, defined as bilirubin levels less than or equal to 20 µmol/l at 6 months of age; and death during the observation interval.

Based on a prospectively maintained Microsoft Excel database, all statistical analyses were performed with statistical computing software R^15^. Means were calculated for normally distributed data and medians for non-normally distributed data. Standard deviations (s.d.) are given for means and interquartile ranges (i.q.r.) for medians. The significance of differences between the three subgroups were calculated using the ANOVA test for normally distributed data (followed by a post-hoc Tukey–Kramer test for multiple comparison), the Kruskal–Wallis test for non-normally distributed data (followed by a post-hoc Bonferroni test for multiple comparison), and the chi-squared test or Fisher’s exact test (if values less than 5) for binary variables. Kaplan–Meier curves were calculated for estimating survival with numbers at risk. These tables were truncated when the number at risk was smaller than one-third of the starting figure. Differences between groups were assessed using a two-tailed Mantel–Cox log rank test at a level of significance of 5 per cent.

## Results

### Study population and baseline patient characteristics

Four hundred and twenty-four patients with BA were treated at Bicêtre, Paris-Saclay University Hospital in the study interval. The early BDS group consisted of 330 patients (78 per cent), the late BDS group consisted of 69 patients (16 per cent) and the no BDS group consisted of 25 patients (6 per cent). Late BDS was performed at a median age of 117 (range 90–198) days. Fifty-four patients in the late BDS group underwent Kasai PE and 15 patients in this group underwent portocholecystostomy or other forms of BDS. Two hundred and sixty-seven of the 330 patients in the early BDS group underwent Kasai PE and 63 patients in this group underwent portocholecystostomy or other forms of BDS. See *Table 1* for patient characteristics in the two groups of late BDS and no BDS.

**Table 1:** Patient characteristics with preoperative and postoperative data for all three subgroups

	Early BDS, *n* = 330	Late BDS, *n* = 69	No BDS, *n* = 25	*P*
Sex ratio (M : F)	145 : 185	27 : 42	10 : 15	0.729
Premature birth	23 (7.0)	13 (19)	5 (20)	0.002 Late *versus* no BDS: 0.900
Associated malformation	30 (9.1)	6 (9)	2 (8)	0.980
Age at BDS/diagnosis (days), median (i.q.r.)	53 (31)	107 (32)	126 (80)	<0.001 Late *versus* no BDS: 0.757
Bilirubin at BDS/diagnosis (µmol/l), median (i.q.r.)	168 (80)	186 (98)	208 (69)	0.009 Late *versus* no BDS: 0.462
AST at BDS/diagnosis (U/l), median (i.q.r.)	230 (141)	267 (192)	318 (284)	0.004 Late *versus* no BDS: 0.600
ALT at BDS/diagnosis (U/l), median (i.q.r.)	175 (123)	176 (150)	177 (254)	0.603
Weight at BDS/diagnosis (g), mean(s.d.)	4080(828)	5080(2469)	5560(751)	<0.001 Late *versus* no BDS: <0.001
Postoperative complications after BDS	83 (25.2)	15 (21)	n/a	0.549
Major complications	14 (4.2)	5 (7)	n/a	0.287
Postoperative mortality after BDS	2 (0.6)	0	n/a	0.970
Alive with native liver at last follow-up	128 (38.8)	18 (26)	0	0.001 Late *versus* no BDS: 0.003
LT during observation interval	165 (50.0)	41 (59)	20 (80)	0.008 Late *versus* no BDS: 0.06
Age at first LT (months), median (i.q.r.)	18 (25)	20 (22)	13 (16)	0.032 Late *versus* no BDS: 0.016
Deaths during observation interval	33 (10.0) On LT waiting list: 15	9 (13) On LT waiting list: 3	5 (20) On LT waiting list: 4	0.262

Values are *n* (%) unless otherwise indicated. Kruskal–Wallis test (with post-hoc Bonferroni test) for non-normally distributed data; ANOVA test (with Tukey–Kramer test) for normally distributed data; chi-squared test/Fisher’s exact test for binary variables. *P* values refer to comparison between all three subgroups unless otherwise specified. BDS, biliary drainage surgery; early BDS, BDS before 90 days of life; late BDS, BDS after 90 days of life; AST, aspartate aminotransferase; ALT, alanine aminotransferase; i.q.r., interquartile range; n/a, not applicable; LT, liver transplantation.

### Postoperative course and outcome of late BDS

Overall postoperative 90-day morbidity of late BDS was 21 per cent (15/69), with major complications occurring in five patients (7 per cent), as compared with an overall 90-day morbidity of 25.2 per cent (86/330) and a rate of major complications of 4.2 per cent (14/330) in the early BDS group. In the late Kasai group, five patients suffered from cholangitis, and six patients suffered from postoperative ascites. Two patients were reoperated on for bile leakage, one of them after portocholecystostomy and one after Kasai PE. In three patients, postoperative ileus due to intussusception/volvulus indicated surgical revision. Success of BDS, defined as serum bilirubin below 20 µmol/l at 6 months of age, was observed in 19 of 69 patients (28 per cent), as compared with a success rate of 55.2 per cent for early BDS (*P* = 0.001). The median age of the patients with success of late BDS was 113 (range 90–158) days. Seven patients (11 per cent) had to undergo LT as infants before turning 12 months. Nine deaths occurred in the late BDS group during the observation interval: two patients died of sepsis and eventual multiple organ failure after re-transplantation; three patients with associated malformations died before listing due to cardiac failure; one patient died of meningitis not associated with BA before listing; and three patients died while being on the waiting list (two of hepatorenal syndrome and one of liver failure). At 2 years, 34 patients in the late BDS group were alive and not transplanted (49 per cent). Eighteen children (26 per cent) were alive with their native liver at last median follow-up of 97 (range 13–185) months.

### Reasons for not performing BDS

In the no BDS group, 18 of 25 patients had manifest portal hypertension and thus did not undergo BDS. In 14 of those 18 patients, diagnosis of portal hypertension was made by ultrasonography. Criteria for diagnosis of portal hypertension via ultrasonography were visible collateral veins in the hepatic hilum, and/or an increased stiffness of the liver and spleen. In four patients, explorative laparotomy revealed visible collateral veins in the hepatic hilum as clear signs of manifest portal hypertension, and no BDS was performed. In four of the 25 patients, advanced age at diagnosis of BA (greater than or equal to 6 months) was the reason for not performing BDS. In one case, co-morbidities contraindicated early surgery, and in one other case the reason was unknown. Five deaths occurred in the no BDS group during the observation interval: one patient with associated malformations died of cardiac failure before being listed for LT; and four patients died while being on the waiting list (one of liver failure and three of hepatorenal syndrome).

### Comparison of native liver and overall survival

**Fig. 1:**
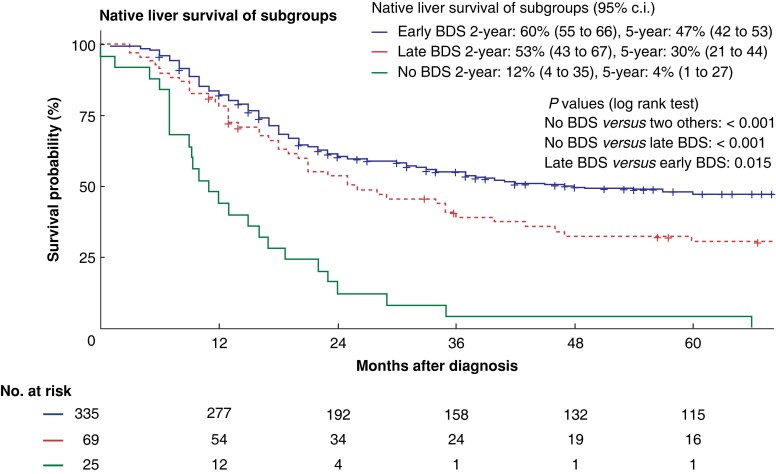
Kaplan–Meier curves for native liver survival of the three subgroups including the numbers at risk and the statistical significance of the difference between all three groups tested with a two-tailed Mantel-Cox log rank test at a level of significance of 5 per cent BDS, biliary drainage surgery; early BDS, BDS before 90 days of life; late BDS, BDS after 90 days of life.

**Fig. 2:**
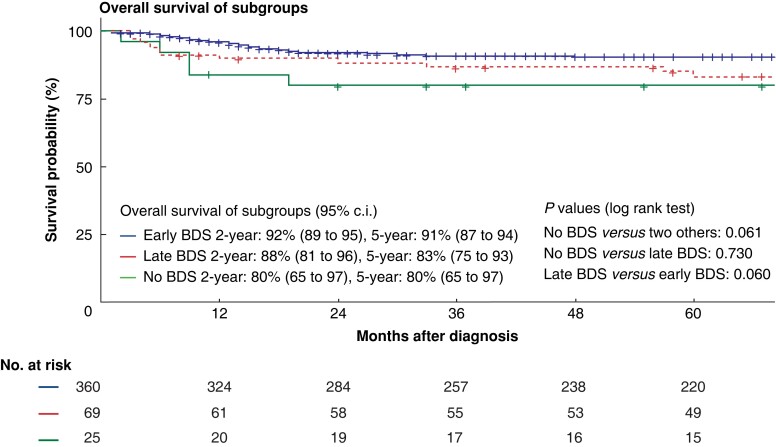
Kaplan–Meier curves for overall survival of the three subgroups including the numbers at risk and the statistical significance of the difference between all three groups tested with a two-tailed Mantel-Cox log rank test at a level of significance of 5 per cent BDS, biliary drainage surgery; early BDS, BDS before 90 days of life; late BDS, BDS after 90 days of life.

The median follow-up of the whole patient cohort was 90 (mean 99, range 1–293) months. Six patients in the late BDS group and three patients in the no BDS group were lost to follow-up between 1 and 60 months after diagnosis (median 10 months). Thirty patients in the early BDS group were lost to follow-up after a median of 15 (range 1–37) months. Two- and 5-year native liver survival among the entire cohort of 424 patients were 57 per cent (95 per cent c.i. 52 to 62 per cent) and 42 per cent (95 per cent c.i. 37 to 47 per cent) respectively. Native liver survival differed significantly among the subgroups (no BDS *versus* late BDS: *P* < 0.001, no BDS *versus* early BDS: *P* < 0.001, late BDS *versus* early BDS: *P* = 0.015, see *Fig. 1*). Whereas patients undergoing late BDS had 2- and 5-year native liver survival of 53 per cent (95 per cent c.i. 43 to 67 per cent) and 30 per cent (95 per cent c.i. 21 to 44 per cent) respectively, patients without BDS had native liver survival of 12 per cent (95 per cent c.i. 4 to 35 per cent) at 2 years, and 4 per cent (95 per cent c.i. 1 to 27 per cent) at 5 years. In comparison, patients undergoing BDS before 90 days had native liver survival of 60 per cent (95 per cent c.i. 55 to 66 per cent) and 47 per cent (95 per cent c.i. 42 to 53 per cent) at 2 and 5 years respectively (*Fig. 2*).

Overall survival was not significantly different across the three subgroups, as shown in *Fig. 2*. However, a trend towards better survival in patients undergoing early BDS was observed, followed by the group of patients with late BDS. Patients without BDS had the lowest 2- and 5-year survival (*Fig. 2*).

## Discussion

Sixteen percent of all patients diagnosed with BA in the authors' tertiary care centre underwent late BDS (greater than 90 days of age). This rate of patients operated on after 90 days is within the range of previous studies that reported on late Kasai procedures in children older than 3 months^5,6,16,17^. The success rate regarding bilirubin clearance of patients with late BDS (28 per cent) in this cohort was significantly lower than in patients with BDS before 90 days of age (55 per cent). This is in line with numerous previous studies, which showed adverse effects of older age at the time of BDS in children with BA^1,2,5,6,18^. On the other hand, several studies reported encouraging success rates in patients undergoing late BDS of up to 55 per cent^16^ and 5-year native liver survival of up to 45 per cent^5^. In this series, the success rate of late BDS was 28 per cent, hence similar to results of other large cohort studies^6,16^. For 88 per cent of the patients undergoing late BDS, LT in infancy could be avoided. More than half of the children in this series who underwent late BDS were alive with their native liver after 2 years, 30 per cent still after 5 years. These results confirm the potential to delay LT in patients with BA even when BDS is performed late. In view of persisting organ shortage, in particular for very small infants with a bodyweight of less than 10 kg, prolongation of native liver survival is highly desirable^11,12^. Moreover, many studies indicated a worse outcome of LT in children with BA under the age of 12 months^19–21^, which reinforces the need for delaying LT and emphasizes the importance of BDS in children aged 3 months or older.

Ninety days has been repeatedly defined as an important threshold in the surgical treatment of children with BA^5,6,22^. Several multicentre or registry studies suggested that the prognosis for native liver survival is significantly worse in patients undergoing BDS after 3 months of life^2,6,23^. However, other large cohort studies did not confirm these findings and no correlation between age at Kasai and postoperative bilirubin clearance was found^24–28^. The association of age at BDS and short- as well as long-term outcome in BA does not seem to be linear^24^. Accordingly, long-term native liver survival has been previously reported in children who were older than 90 days at the time BDS was performed^17,23,24^. As reported by other studies, long-term native liver survival was achieved in several patients, even when they were operated after 120 days of life also in this series. This finding further strengthens the indication of BDS after 3 months of life. The direct perioperative risks of BDS in general, and late BDS in particular, are low, as shown by a postoperative mortality rate of 0 per cent and a major complication rate of 7 per cent in the current cohort. Within the subgroup of late BDS, there was no tendency towards worse outcome with increasing age, a finding that further questions age as a single decisive factor in those patients. At the same time, BDS did not seem to have negative consequences for possible consecutive LT, which is mirrored by the superior overall survival of patients with late BDS compared with those with no BDS in this study. While there are contradictory results concerning the ramifications of BDS on surgical risks of LT and post-transplant outcome in children with BA^7,8^, recent studies on the impact of previous Kasai operation *versus* primary LT concluded that post-LT outcome is at least equal in those two groups, with a significantly higher age at LT in the Kasai group^9,12^. The authors' findings reveal that age alone should probably not be a criterion to decide against BDS and that other factors must be taken into account, some of which might not have been identified yet.

This study has several limitations, including the retrospective, non-randomized design. However, it should be evaluated in the context of a rare paediatric condition with small patient numbers, where evidence from RCTs is lacking and retrospective trials are an invaluable resource to generate evidence. Moreover, the two groups of late BDS and no BDS were not matched because of the limited sample size and therefore inevitably exhibit baseline differences. Although preoperative bilirubin and liver aminotransferase levels did not differ significantly between the late BDS and no BDS subgroups, the high percentage of manifest portal hypertension in the no BDS group may hint at more advanced stages of disease in these children compared with the late BDS group, which in turn would weaken the comparability of long-term outcomes. A major strength of this study is the comparatively large number of patients, and, to the best of the authors' knowledge, it is the largest single-centre series of patients undergoing late BDS. This allowed for a meaningful comparative study of contemporary subgroups. Moreover, BA patients are treated from first diagnostics to potential LT including long-term follow-up at the authors' institution, and one specialized dedicated surgical team performs BDS as well as paediatric LT. As a consequence, care of these patients remains in one hand, and bias towards one approach is strongly reduced in this study, as there is no personal or institutional interest in advocating either late BDS or primary LT in patients with a delayed diagnosis of BA.

These results support the recommendation to also consider BDS in patients older than 90 days. Although the indication for surgery in these patients must be thoroughly assessed and remains an individual decision, advanced infant age alone is not an appropriate criterion to deny these patients BDS and thus the chance of long-term native liver survival. Future studies should investigate additional factors that predict the outcome of patients with late diagnosis of BA to advance towards an individually tailored therapy. In addition, the impact of late BDS on intraoperative and postoperative complications of secondary LT should be further investigated.

## Data Availability

The datasets used and/or analysed during the current study are available from the corresponding author upon reasonable request.

## References

[1] Fanna M , Masson G, Capito C, Girard M, Guerin F, Hermeziu B et al Management of biliary atresia in France 1986 to 2015: long-term results. J Pediatr Gastroenterol Nutr 2019;69:416–424 31335841 10.1097/MPG.0000000000002446

[2] Serinet M-O , Wildhaber BE, Broue P, Lachaux A, Sarles J, Jacquemin E et al Impact of age at Kasai operation on its results in late childhood and adolescence: a rational basis for biliary atresia screening. Pediatrics 2009;123:1280–1286 19403492 10.1542/peds.2008-1949

[3] Okubo R , Nio M, Sasaki H, Society JBA. Impacts of early Kasai portoenterostomy on short-term and long-term outcomes of biliary tresia. Hepatol Commun 2021;5:234–243 33553971 10.1002/hep4.1615 PMC7850309

[4] Davenport M , Caponcelli E, Livesey E, Hadzic N, Howard E. Surgical outcome in biliary atresia: etiology affects the influence of age at surgery. Ann Surg 2008;247:694–698 18362634 10.1097/SLA.0b013e3181638627

[5] Davenport M , Puricelli V, Farrant P, Hadzic N, Mieli-Vergani G, Portmann B et al The outcome of the older (≥100 days) infant with biliary atresia. J Pediatr Surg 2004;39:575–581 15065031 10.1016/j.jpedsurg.2003.12.014

[6] Chardot C , Carton M, Spire-Bendelac N, Le Pommelet C, Golmard J-L, Reding R et al Is the Kasai operation still indicated in children older than 3 months diagnosed with biliary atresia? J Pediatr 2001;138:224–228 11174620 10.1067/mpd.2001.111276

[7] Superina R . Biliary atresia and liver transplantation: results and thoughts for primary liver transplantation in select patients. Pediatr Surg Int 2017;33:1297–1304 29030698 10.1007/s00383-017-4174-4

[8] Alexopoulos SP , Merrill M, Kin C, Matsuoka L, Dorey F, Concepcion W et al The impact of hepatic portoenterostomy on liver transplantation for the treatment of biliary atresia: early failure adversely affects outcome. Pediatr Transplant 2012;16:373–378 22463739 10.1111/j.1399-3046.2012.01677.x

[9] Lemoine CP , LeShock JP, Brandt KA, Superina R. Primary liver transplantation vs. transplant after Kasai portoenterostomy for infants with biliary atresia. J Clin Med 2022;11:3012 35683401 10.3390/jcm11113012 PMC9181323

[10] Uecker M , Kuebler JF, Schukfeh N, Pfister ED, Baumann U, Petersen C et al Kasai procedure in patients older than 90 days: worth a cut. Eur J Pediatr Surg 2022;32:80–84 34918313 10.1055/s-0041-1740556

[11] Neto JS , Feier FH, Bierrenbach AL, Toscano CM, Fonseca EA, Pugliese R et al Impact of Kasai portoenterostomy on liver transplantation outcomes: a retrospective cohort study of 347 children with biliary atresia. Liver Transpl 2015;21:922–927 25832004 10.1002/lt.24132

[12] Yoeli D , Choudhury RA, Sundaram SS, Mack CL, Roach JP, Karrer FM et al Primary vs. salvage liver transplantation for biliary atresia: a retrospective cohort study. J Pediatr Surg 2022;57:407–413 35065808 10.1016/j.jpedsurg.2021.12.027

[13] Von Elm E , Altman DG, Egger M, Pocock SJ, Gøtzsche PC, Vandenbroucke JP et al The Strengthening the Reporting of Observational Studies in Epidemiology (STROBE) statement: guidelines for reporting observational studies. Int J Surg 2014;12:1495–1499 25046131 10.1016/j.ijsu.2014.07.013

[14] Dindo D , Demartines N, Clavien PA. Classification of surgical complications: a new proposal with evaluation in a cohort of 6336 patients and results of a survey. Ann Surg 2004;240:205–213 15273542 10.1097/01.sla.0000133083.54934.ae PMC1360123

[15] R Core Team . R: A Language and Environment for Statistical Computing. Vienna, Austria: R Foundation for Statistical Computing, 2021

[16] Wong K , Chung P, Chan I, Lan L, Tam P. Performing Kasai portoenterostomy beyond 60 days of life is not necessarily associated with a worse outcome. J Pediatr Gastroenterol Nutr 2010;51:631–634 20818266 10.1097/MPG.0b013e3181e8e194

[17] Ramachandran P , Safwan M, Tamizhvanan V, Balaji MS, Unny AK, Vij M et al Age is not a criterion in patient selection for Kasai portoenterostomy. J Indian Assoc Pediatr Surg 2019;24:271 31571758 10.4103/jiaps.JIAPS_182_18 PMC6752066

[18] Altman RP , Lilly JR, Greenfeld J, Weinberg A, van Leeuwen K, Flanigan L. A multivariable risk factor analysis of the portoenterostomy (Kasai) procedure for biliary atresia: twenty-five years of experience from two centers. Ann Surg 1997;226:348–353 9339941 10.1097/00000658-199709000-00014 PMC1191037

[19] Fouquet V , Alves A, Branchereau S, Grabar S, Debray D, Jacquemin E et al Long-term outcome of pediatric liver transplantation for biliary atresia: a 10-year follow-up in a single center. Liver Transpl 2005;11:152–160 15666395 10.1002/lt.20358

[20] Barshes NR , Lee TC, Balkrishnan R, Karpen SJ, Carter BA, Goss JA. Orthotopic liver transplantation for biliary atresia: the U. S. experience. Liver Transpl 2005;11:1193–1200 16184564 10.1002/lt.20509

[21] Arnon R , Annunziato RA, D’Amelio G, Chu J, Shneider BL. Liver transplantation for biliary atresia: is there a difference in outcome for infants? J Pediatr Gastroenterol Nutr 2016;62:220–225 26418214 10.1097/MPG.0000000000000986

[22] Nio M , Wada M, Sasaki H, Tanaka H. Effects of age at Kasai portoenterostomy on the surgical outcome: a review of the literature. Surg Today 2015;45:813–818 25212566 10.1007/s00595-014-1024-z

[23] Lykavieris P , Chardot C, Sokhn M, Gauthier F, Valayer J, Bernard O. Outcome in adulthood of biliary atresia: a study of 63 patients who survived for over 20 years with their native liver. Hepatology 2005;41:366–371 15660386 10.1002/hep.20547

[24] Shinkai M , Ohhama Y, Take H, Kitagawa N, Kudo H, Mochizuki K et al Long-term outcome of children with biliary atresia who were not transplanted after the Kasai operation: >20-year experience at a children's hospital. J Pediatr Gastroenterol Nutr 2009;48:443–450 19330933 10.1097/mpg.0b013e318189f2d5

[25] Leonhardt J , Kuebler J, Leute P, Turowski C, Becker T, Pfister E-D et al Biliary atresia: lessons learned from the voluntary German registry. Eur J Pediatr Surg 2011;21:82–87 21157692 10.1055/s-0030-1268476

[26] Schneider , Brown M., Haber B., Whitington P.F., Schwarz K., Squires R et al Biliary Atresia Research Consortium. A multicenter study of the outcome of biliary atresia in the United States, 1997 to 2000. J Pediatr 2006 148:467–474 16647406 10.1016/j.jpeds.2005.12.054

[27] Wildhaber BE , Coran AG, Drongowski RA, Hirschl RB, Geiger JD, Lelli JL et al The Kasai portoenterostomy for biliary atresia: a review of a 27-year experience with 81 patients. J Pediatr Surg 2003;38:1480–1485 14577071 10.1016/s0022-3468(03)00499-8

[28] McKiernan PJ , Baker AJ, Kelly DA. The frequency and outcome of biliary atresia in the UK and Ireland. Lancet 2000;355:25–29 10615887 10.1016/S0140-6736(99)03492-3

